# Salt tolerance during germination and seedling growth of wild wheat *Aegilops tauschii* and its impact on the species range expansion

**DOI:** 10.1038/srep38554

**Published:** 2016-12-08

**Authors:** Daisuke Saisho, Shigeo Takumi, Yoshihiro Matsuoka

**Affiliations:** 1 Institute of Plant Science and Resources, Okayama University, Kurashiki 710-0046, Japan; 2 Laboratory of Plant Genetics, Graduate School of Agricultural Science, Kobe University, Nada-ku, Kobe 657-8501, Japan; 3 Fukui Prefectural University, Matsuoka, Eiheiji, Yoshida, Fukui 910-1195, Japan

## Abstract

Adaptation to edaphic stress may have a key role in plant species range expansion. *Aegilops tauschii* Coss., the common wheat’s D-genome progenitor native to the Transcaucasus-Middle East region, is a good model to study the relationships between soil salinity and plant distributions: one of its intraspecific sublineages, TauL1b, drove the long-distance eastward expansion of this species range reaching semi-arid-central Asia. Salt tolerance during germination and seedling growth was evaluated in 206 *Ae. tauschii* accessions by treating seeds with NaCl solutions differing in concentrations. Differences in natural variation patterns were analyzed between sublineages and associated with natural edaphic condition variables, and then compared with reproductive trait variation patterns. The natural variations observed in NaCl-induced-stress tolerance had clear geographic and genetic structure. Seedling growth significantly increased in the TauL1b accessions that were collected from salt-affected soil habitats, whereas germinability did not. Principal component analysis suggested that the NaCl-induced-stress tolerances and reproductive traits might have had a similar degree of influence on *Ae. tauschii*’s eastward range expansion. Adaptation to salt-affected soils through increased seedling growth was an important factor for the species’ successful colonization of the semi-arid central Asian habitats. TauL1b accessions might provide useful genetic resources for salt-tolerant wheat breeds.

Species ranges expand through individuals’ dispersal to novel habitats and/or adaptation to local environmental conditions. These individuals face several environmental challenges to their survival and propagation in the new habitats, including extreme climatic and edaphic conditions, unusual competition, predation, and disease^1^. Foreign abiotic and biotic stresses can cause plastic phenotypic changes in the migrants and in their early descendants and, eventually, result in the evolution of local adaptation in the following generations through genetic changes. Peripheral populations of geographically widespread species are of great evolutionary and ecological interest because they represent model systems to study the genetic mechanisms underlying local adaptation^2^.

Phenotypic changes in ecologically functional traits can be essential to species range expansion through adaptation to local environments. Several studies emphasized the importance of reproductive traits change in the evolution of plant local adaptation^3^. In general, adaptive phenotypic changes in the new habitats can occur rapidly within tens of generations, particularly when the species’ standing genetic variation can provide alleles that would be favored under the new environmental conditions^4^,^5^. Climate conditions, such as temperature, precipitation, and photoperiod, are common environmental factors that can cause adaptive phenotypic changes^6^,^7^. However, in the process of species range expansion, migrating individuals/populations may have to overcome several stressful abiotic factors other than climate conditions in order to survive and propagate in the new habitats. Edaphic stress is one of the key abiotic factors that may serve as a selective agent in the evolution of local adaptation^8^,^9^. In natural settings, plants’ germination, vegetative growth, root and aboveground architecture, metabolism, and reproduction may be strongly influenced by soil’s shallowness and poor drainage (physical conditions), as well as water and nutrient deficiency, acidity, alkalinity, salinity, and metal toxicity (chemical conditions). For this reason, edaphic factors might potentially explain the patterns of local adaptation. Nevertheless, relatively little is known about how edaphic stress functions as a selective agent in ecological and evolutionary contexts. Particularly, much remains to be studied about the relationships between soil salinity and plant distributions: soil chemical variables considered in biogeographic studies are usually limited to soil moisture/texture, pH, and nutrients^10^.

This paper reports an ecogeographic analysis of the natural variation occurring during germination and early seedling growth of the wild wheat *Aegilops tauschii* Coss. under NaCl-induced stress. *Ae. tauschii* (formerly known as *Aegilops squarrosa* L.) is the D-genome progenitor of hexaploid common wheat *Triticum aestivum* L.^11^,^12^. *Ae. tauschii* weedy-stands often occur in wheat and barley fields within the species range^13^, but the species natural habitats encompass sandy seashores, deserts margins, stony hills, steppes, wastelands, roadsides, and humid temperate forests. It is a polymorphic self-pollinating diploid species with a wide geographic range in central Eurasia. The center of *Ae. tauschii*’s distribution is in the southern coastal region of the Caspian Sea and Azerbaijan. From the center, the species range spreads eastward, to Pakistan and western China, via the Kopet Dag Mountains of Turkmenistan, and westward, to central Syria, via the southeastern Turkey valleys^14^,^15^. The biogeography of *Ae. tauschii* is intriguing. The genus *Aegilops* has, in addition to 12 polyploid species, 10 diploid species that are thought to have radiated some million years ago in the Transcaucasus. *Ae. tauschii*’s geographic distribution differs from that of its congeners, as this diploid species is the only one whose distribution range expanded eastward, i.e., to the Asian part of the continent; all the other diploid species migrated toward the west and southwest of the Transcaucasus. *Ae. tauschii* is the only currently known *Aegilops* species that naturally grows in China^14^,^15^.

It is not clear why and how *Ae. tauschii* made its way to central Asia, while the other diploid species expanded their ranges westward from Transcaucasus. The central Asian part of *Ae. tauschii*’s distribution range is characterized by cold semi-arid and continental climate conditions, whereas the Transcaucasus-Middle East part is largely under temperate climate conditions^16^. Accordingly, *Ae. tauschii* intraspecific lineages and sublineages are potentially useful to study the ecological and evolutionary association between soil salinity tolerance patterns and species distribution ranges in plants inhabiting dry lands. Thus, this study aimed to: (1) provide a broad picture of the natural variation patterns in *Ae. tauschii*’s NaCl-induced-stress tolerance during its germination and early seedling growth, (2) clarify if the species’ eastward range expansion is associated with an adaptive phenotypic change in salt tolerance by comparing the natural variation patterns of lineages and sublineages in various edaphic conditions, and (3) provide insights on the importance of salt tolerance during germination and seedling growth for *Ae. tauschii*’s eastward range expansion.

## Materials and Methods

### Study system

Based on the patterns of molecular marker variations, three genetic lineages were defined for *Ae. tauschii*: two major lineages (TauL1 and TauL2), each including two distinctive sublineages (TauL1a and TauL1b within TauL1; TauL2a and TauL2b within TauL2), and one minor lineage (TauL3)^17^,^18^,^19^,^20^ (Supplementary Fig. S1). Of these lineages, TauL2 and TauL3 are genetically closer to the common wheat D genome than TauL1. Geographically, TauL1 has a wide distribution range spanning from central Syria to western China, whereas TauL2 and TauL3 are restricted to the Transcaucasus-Middle East region and Georgia, respectively (Supplementary Fig. S2). Interestingly, TauL1b sublineage, which derived from TauL1a, is the only sublineage occurring in the central Asian part of the species range, suggesting that TauL1b drove *Ae. tauschii*’s eastward range expansion^19^. A population genetic analysis showed that the TauL1b accessions were composed of two large and one small genepools and that they could be classified into three genetic groups: (1) the accessions having alleles mostly (>50%) derived from one of the large gene pools (LowQ2); (2) the accessions having alleles mostly (>50%) derived from the other large gene pool (HighQ2); (3) the accessions having alleles almost exclusively derived from the small gene pool (Q3) (Supplementary Fig. S1).

### Plant materials

Two hundred and six *Ae. tauschii* Coss. accessions representing the entire natural habitat range and adventive populations in the Shaanxi and Henan provinces of China were used (Supplementary Table S1). These accessions were selected because their natural variation patterns in several reproductive and morphological traits, as well as their molecular marker genotypes, had been characterized in detail^19^. Only five accessions represented the TauL3 lineage. Despite that the sample size was small, we included the TauL3 accessions to draw a species-wide picture of the natural variation patterns in *Ae. tauschii*’s NaCl-induced-stress tolerance during its germination and early seedling growth. For each accession, we used seeds propagated by selfing from a single plant that flowered in a greenhouse during spring. Seeds were stored at 4 °C for 10–15 months before use.

### Evaluation of salt tolerance during germination and seedling growth

Seeds of each accession were treated with 0.5% (w/v; 85.5 mM), 1.0% (w/v; 171 mM), and 1.5% (w/v; 257 mM) NaCl solutions and with distilled water (as a control treatment). These NaCl concentrations were chosen because they were shown to be useful to study the salt tolerance during germination and seedling growth in wild *Hordeum* species that often occur together with *Ae. tauschii* in natural habitats^21^. The electrical conductivity of 0.5%, 1.0%, and 1.5% NaCl solutions roughly corresponded to 8.6 dS/m, 17.0 dS/m, and 26.0 dS/m, respectively. Nine seeds from each accession were used in each treatment, except for the 0.5% NaCl treatment of KU-2111 and the 1.0% NaCl treatment of KU-2042 (eight seeds were used in both cases). For each accession, the seeds were placed on a sand layer (ca. 15 mm thick), moistened with a 0.5%, 1.0%, or 1.5% NaCl solution (or distilled water in the case of controls), and set at the bottom of a plant tissue culture container. After two weeks at 4 °C under dark condition, germination and seedling growth took place at 22 °C under a 12/12 h light/dark photoperiod for another two weeks. To reduce the effect of lurking environmental condition differences on phenotypes, the containers were arranged randomly in a growth chamber. Two traits, germinability and seedling growth ability, were measured to evaluate salt tolerance during early development under a given treatment condition. Germinability was determined by counting the number of germinated seeds in a container (germinated seed number, abbreviated as GN hereafter), whereas the seedling growth ability was calculated as the mean length of the first leafs emerging from germinated seeds (mean length, abbreviated as ML hereafter). Seeds with a coleoptile shorter than 5.0 mm were considered non-germinated and excluded from ML calculation.

### Edaphic and phenotypic datasets

Because the information on the edaphic conditions of original habitats was not available, we used the soil property estimates that were obtained from two sources: the 30 by 30 arcsec (roughly 1 km cell) resolution salinity (electrical conductivity) dataset from the Harmonized World Soil Database ver. 1.2^22^ and the 30 by 30 arcsec resolution available water capacity dataset from the World soil property estimates for broad-scale modeling (WISE30sec)^23^. The Harmonized World Soil Database combined regional and national updates of soil information worldwide, whereas WISE30sec provided a dataset that was created using the soil map unit delineations of the Harmonized World Soil Database overlaid by soil property estimates derived from analyses of the ISRIC-WISE soil profile database. ArcGIS ver. 10 (ESRI) was used to extract the estimates from these sources based on the geographic coordinates of the sampling sites. For the 20 Caspian coastal habitats, salinity estimates and/or available water capacity estimates were extracted based on the locality information. The *Ae. tauschii* flowering time (i.e., days from coleoptile emergence to flower)^24^ and number of spikelets per spike^19^ datasets were taken from previous studies.

### Statistical analysis

Statistical calculations were performed using the R Commander software ver. 2.1 for R ver. 3.2 (The R Foundation for Statistical Computing Platform)^25^ (for Fisher’s exact test), the R package ‘ggplot2’^26^ (for data plotting), the R package ‘lawstat’^27^ (for Brunner-Munzel test), the R package ‘ecodist’^28^ (for dissimilarity matrix calculation and partial Mantel test), JMP ver. 12.1 (SAS Institute Inc.) (for Welch’s *t* test, Steel-Dwass test, principal component analysis (PCA), Tukey-Kramer test, and regression), and SPSS ver. 23 (IBM) (for Dunnet T3 multiple comparisons). Dunnet T3 multiple comparisons were used when the variances in the groups were unequal. Partial Mantel tests were done using phenotypic, genetic, and latitudinal dissimilarity (distance) matrices with 1,000 permutations. The Bayesian Information Criterion was used for model selection in stepwise multiple regression. GenAlEx ver. 6.5 was used to calculate genetic distances^29^ and pairwise *F*_st_ values^30^,^31^.

## Results

### Natural variation in NaCl-induced-stress tolerance and its association with habitat edaphic conditions

**Figure 1:**
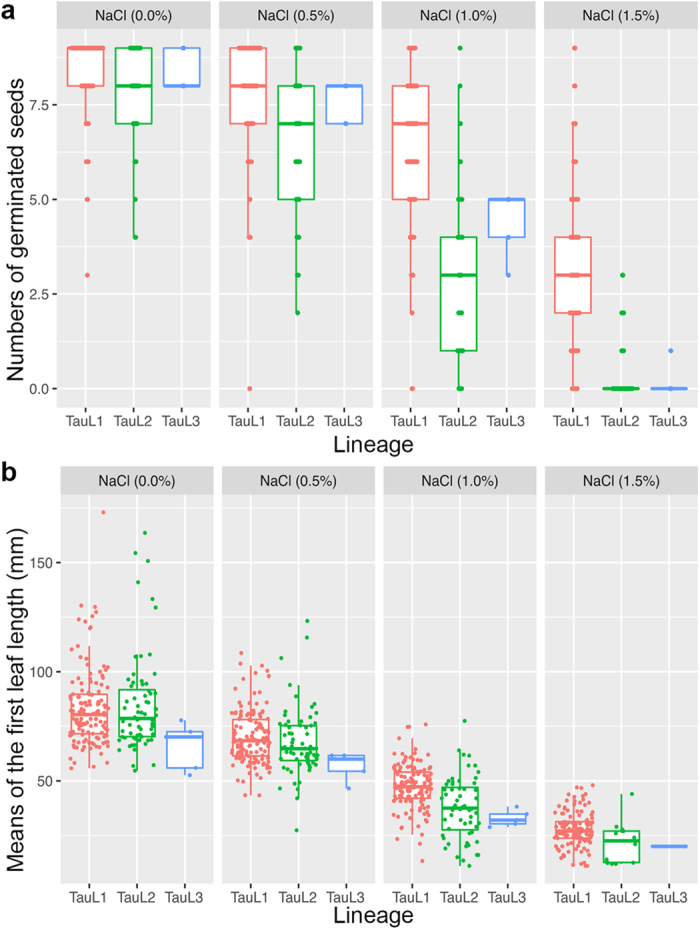
Natural variation of *Ae. tauschii* salt-stress tolerance under experimental NaCl conditions. (**a**) Lineage-wise box and dot plots of germinability (i.e., the number of germinated seeds per accession). (**b**) Lineage-wise box and dot plots of seedling growth ability (i.e., the mean length of the first leaf per accession).

Responses to NaCl-induced stress varied greatly among the tested accessions, albeit stress apparently having a negative overall effect on germination and seedling growth. As the stress increased, both GNs and MLs markedly decreased (Fig. 1). However, GN mean values were larger in TauL1 than in TauL2 and TauL3 in all NaCl treatments (Supplementary Table S2). Because GNs (count data having a maximum value) were not well modeled by a normal distribution, we used Brunner-Munzel test of stochastic equality to examine if the GNs values were statistically different between the lineages. As the result, a significant difference between the TauL1 and TauL2 GN values was found in each treatment (*P* < 0.01). In the 1.5% NaCl treatment, 119 TauL1 accessions out of the 133 (89.5%) had at least one germinated seed (i.e., they showed seedling growth ability), whereas only 20.6% (14 out of 68) of the TauL2 accessions and 20.0% (one out of five) of the TauL3 accessions showed seedling growth ability. Furthermore, the TauL1 accessions outperformed the TauL2 and TauL3 accessions in seedling growth ability under all NaCl stress conditions, as revealed by TauL1 larger ML mean values compared to TauL2 and TauL3 (Supplementary Table S2). Differences between TauL1 and TauL2 mean ML values were statistically significant in the 1.0% and 1.5% NaCl treatments (Welch’s *t* test). All these findings showed that *Ae. tauschii*’s NaCl-induced-stress tolerance natural variations have a clear genetic structure and that the geographically widespread TauL1 lineage is distinct from the others with respect to under-stress germinability and seedling growth ability.

**Figure 2:**
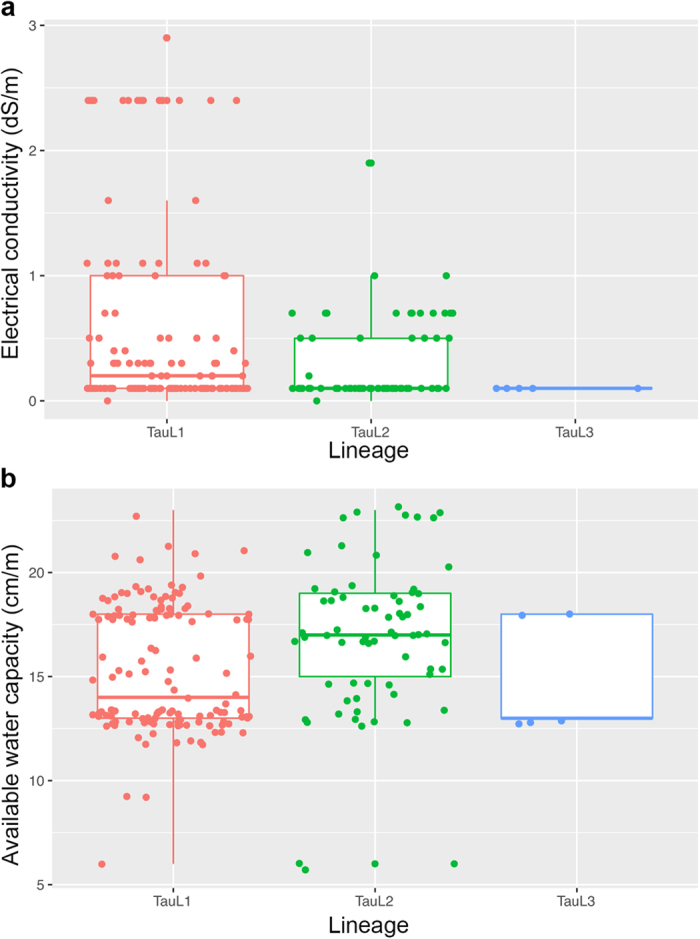
Edaphic conditions of *Ae. tauschii* accessions habitats. (**a**) Lineage-wise box and dot plots of soil salinity (electrical conductivity). The number of plotted habitats is 124 for TauL1 (nine missing), 66 for TauL2, and five for TauL3. Two TauL2 habitats that had very high electrical conductivity values (5.9 dS/m) were not shown. (**b**) Lineage-wise box and dot plots of available water capacity of habitat soils. The number of plotted accessions plotted is 132 for TauL1 (one missing), 68 for TauL2, and five for TauL3.

In general, increases in environmental NaCl concentrations may cause osmotic stress that disturbs plants’ water uptake, as well as ionic stress that interferes with metabolic processes through the activity of excess Na^+^ and Cl^−^ 32. As so, we compared soil salinity and available water capacity conditions between TauL1, TauL2, and TauL3 habitats. Soil salinity estimates were available for the habitats of 197 out of the 206 accessions analyzed, whereas available water capacity estimates were available for 205 accessions (Supplementary Table S1). Several soils of the TauL1 habitats had salinity (i.e., electrical conductivity) values >1 dS/m, whereas salinity value ranges widely overlapped between lineages (Fig. 2a). The soils of two Caspian coastal TauL2 habitats had high electrical conductivity values (5.9 dS/m). Electrical conductivity mean values were larger in TauL1 (0.60 dS/m) than in TauL2 (0.46 dS/m) and TauL3 (0.10 dS/m). A Brunner-Munzel test showed a significant stochastic difference between the TauL1 and TauL2 electrical conductivity values (*P* = 0.00). The ranges of soil available water capacity values also widely overlapped between lineages, but the proportion of low available water capacity (<15 cm/m) habitats (i.e., high osmotic stress habitats) was higher in TauL1 (67/132, 51%) than in TauL2 (15/68, 22%) (Fig. 2b). The proportion was 3/5 (60%) in TauL3. Available water capacity mean values were larger in TauL2 (16.9 cm/m) than in TauL1 (15.3 cm/m) and TauL3 (15.0 cm/m). A Brunner-Munzel test showed a significant stochastic difference between the TauL1 and TauL2 available water capacity values (*P* < 0.00). These findings suggested an association between TauL1 accessions’ increased NaCl-induced-stress tolerances and the edaphic conditions of their natural habitats.

### Increased seedling growth in TauL1b

To address if *Ae. tauschii*’s eastward range expansion is associated with an adaptive change in salt tolerance, the NaCl-induced-stress tolerances of the TauL1 sublineages/groups were compared. To simplify the NaCl-induced-stress tolerance comparisons, a PCA based on the among-accession covariance matrix was performed to produce a single virtual trait (i.e., the first principal component that defined the axis of maximum phenotypic divergence among the accessions in a multivariate space) for each under-stress germinability (based on the GNs under 0.5%, 1.0%, and 1.5% NaCl conditions) and early seedling growth (based on the MLs under 0.5% and 1.0% NaCl conditions). The ML dataset under the 1.5% NaCl treatment was not used due to the large number of accessions (72 in total) not showing the trait. In addition, two accessions, KU-2001 and KU-2003, were excluded from the PCA for under-stress early seedling growth, due to germination failure under 0.5% and/or 1.0% NaCl conditions. The under-stress germinability PCA’s PC1 (named the TauL1-germinability PC) and the under-stress early seedling growth ability PCA’s PC1 (named the TauL1-seedling PC) explained 61.7% and 79.8% of the total variance, respectively. All eigenvectors of both under-stress PCs were positive: 0.24 (GN, 0.5% NaCl, PC loading = 0.42), 0.61 (GN, 1.0% NaCl, PC loading = 0.81), and 0.76 (GN, 1.5% NaCl, PC loading = 0.89) for the TauL1-germinability PC; 0.81 (ML, 0.5% NaCl, PC loading = 0.94) and 0.59 (ML, 1.0% NaCl, PC loading = 0.82) for the TauL1-seedling PC. Accordingly, the accessions’ TauL1-germinability PC and TauL1-seedling PC values provided useful phenotypic indices that positively correlated with the levels of the NaCl-induced-stress tolerances in TauL1.

**Figure 3:**
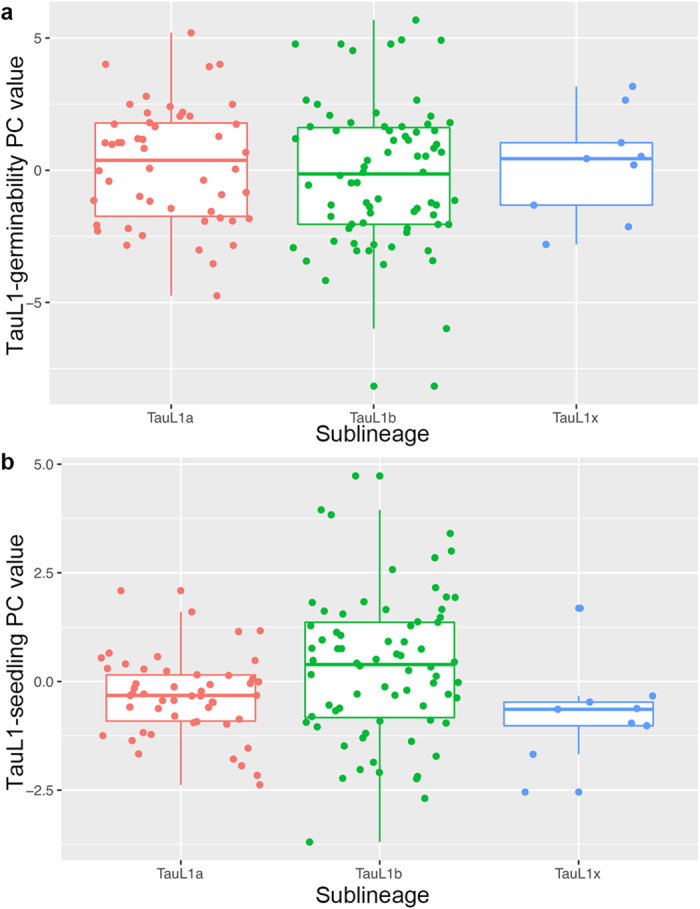
Comparisons of TauL1-germinability PC and TauL1-seedling PC values in TauL1a, TauL1b, and TauL1x. (**a**) Box and dot plots of TauL1-germinability PC values. (**b**) Box and dot plots of TauL1-seedling PC values.

Mean TauL1-germinability PC values were not significantly different between TauL1a, TauL1b, and TauL1x (Tukey-Kramer test, *P* > 0.05) (Fig. 3; Supplementary Table S3). In contrast, mean TauL1-seedling PC values were larger in TauL1b (0.33) than in TauL1a (−0.35) and TauL1x (−0.73), the difference between TauL1a and TauL1b values was significant (Dunnett T3 multiple comparisons, *P* = 0.013). Control (distilled water) experiments showed that the differences of mean germinability values of TauL1a, TauL1b, and TauL1x were not statistically significant (Supplementary Fig. S3; Supplementary Table S4). ML mean values of these sublineages/group varied, but only TauL1a (78.2 mm) and TauL1b (87.4 mm) MLs were significantly different (Dunnett T3 multiple comparisons, *P* = 0.004), indicating that seedling growth was increased in TauL1b relative to TauL1a under control conditions. These results showed that seedling growth ability was larger in TauL1b than in TauL1a under NaCl-induced-stress conditions as well as under NaCl-free condition, whereas germinability was not markedly different.

### Structure of the TauL1b under-stress seedling growth variation

**Figure 4:**
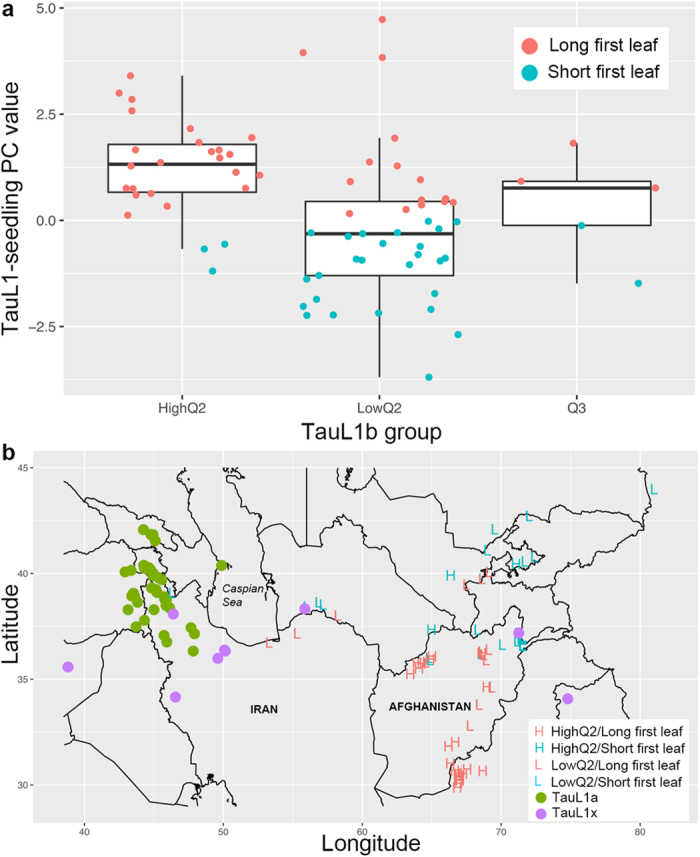
Genetic and geographic structure of the TauL1-seedling PC variation in TauL1b. (**a**) Box and dot plots of the TauL1-seedling PC values in HighQ2, LowQ2, and Q3 groups. For each accession, seedling phenotype is colored according to the key. (**b**) Geographic distribution of HighQ2 and LowQ2 accessions. For each accession, seedling phenotype is colored according to the key. Distributions of the TauL1a and TauL1x accessions are also shown. The map was drawn using the R software [R Core Team, R Foundation for Statistical Computing, Vienna, Austria, R: A Language and Environment for Statistical Computing, (2016) https://www.R-project.org (Date of access 01/03/2016)], the R package ‘ggplot2’ (R version 3.3.0)^26^ [Wickham, H., ggplot2: Elegant Graphics for Data Analysis. (2009) http://ggplot2.org (Date of access 01/03/2016)] and a spatial dataset obtained from DIVA-GIS (version 7.5.0) [Hijmans, R. J., Guarino, L. & Mathur, P., DIVA-GIS., (2012) http://www.diva-gis.org/Data (Date of access: 01/03/2016)].

To study the genetic structure of TauL1b’s under-stress seedling growth variation, we compared TauL1-seedling PC values between HighQ2, LowQ2, and Q3 groups. Within-group mean genetic distances were 104.1 (HighQ2), 128.2 (LowQ2), and 13.6 (Q3) and pairwise *F*_ST_ values showed low to moderate degrees of genetic differentiation between these groups (Supplementary Table S5). Within TauL1b, mean TauL1-seedling PC values were larger in HighQ2 (1.23) than in LowQ2 (−0.25) and Q3 (0.38) and the difference between HighQ2 and LowQ2 means was significant (Turkey-Kramer test, *P* < 0.05) (Fig. 4; Supplementary Table S3). The under-stress TauL1b seedling growth phenotypes were classified into two groups: long first leaf (TauL1-seedling PC values ≥ 0) and short first leaf (TauL1-seedling PC values < 0). In HighQ2, 23 out of 26 accessions (88.5%) had long first-leaf phenotypes. This proportion was lower in LowQ2 (15/41, 36.6%) and in Q3 (3/5, 60.0%). A partial Mantel test showed that phenotypically similar TauL1b accessions had similar genetic compositions, whereas dissimilar accessions had dissimilar genetic compositions, when the effect of latitudinal distances was controlled (Mantel *r* statistic = 0.12, *P* = 0.00). Most long first-leaf accessions (37/41, 90.2%) occurred in the southern portion of TauL1b’s geographic range (latitude < 37.5°). In contrast, the proportion of short first-leaf accessions in northern (13/31, 42.0%) and south (18/31, 58.1%) habitats were comparable (Supplementary Table S6). No significant association was found between the phenotypic and latitudinal dissimilarity (distance) matrices, when the effect of genetic dissimilarity was controlled (partial Mantel test, Mantel *r* statistic = 0.01, *P* = 0.41). Overall, the distributions of long first-leaf and short first-leaf accessions were significantly biased between north and south (Fisher’s exact test, *P* = 0.00) and among TauL1b groups (Fisher’s exact test, *P* < 0.00) (Fig. 4b). These findings showed that under-stress TauL1b seedling growth variations had clear genetic and geographic structures.

### Ecological functions of NaCl-induced-stress tolerance during seedling growth in TauL1b

**Figure 5:**
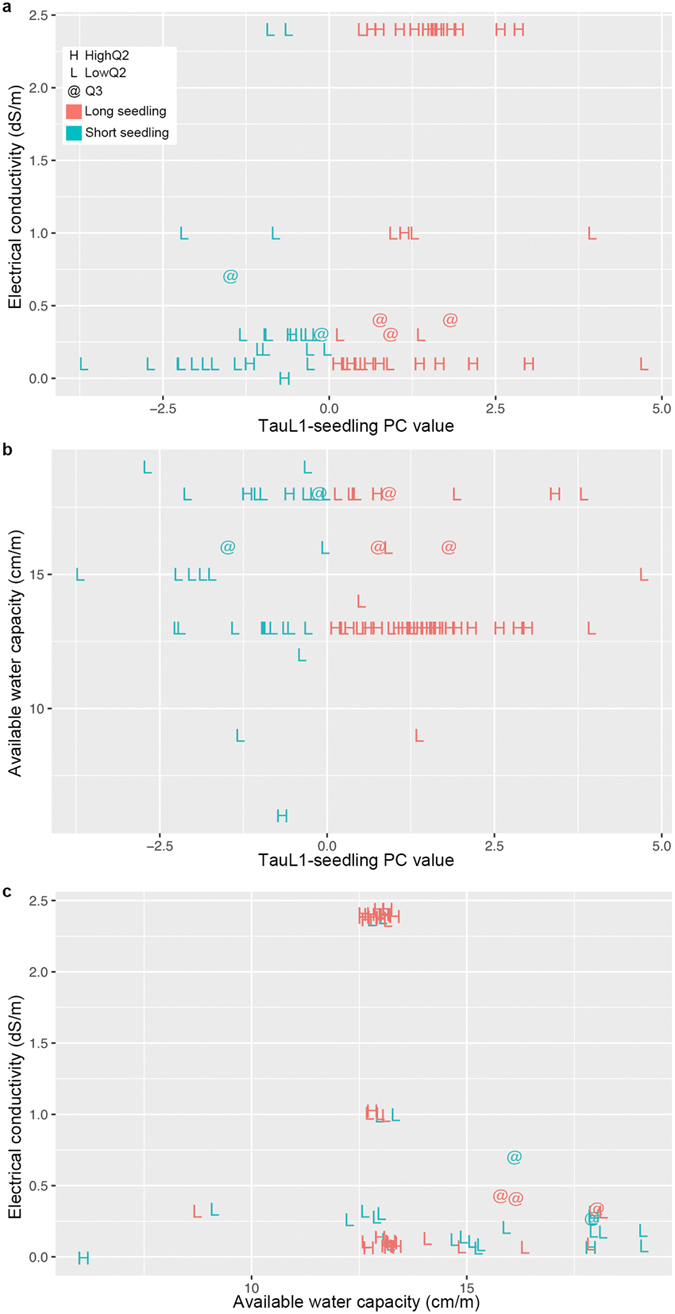
Association of the seedling phenotypes with habitat edaphic conditions in 72 TauL1b accessions. (**a**) Relationship between TauL1-seedling PC (*x*) and soil electrical conductivity (*y*). Eight accessions are not plotted due to missing values. (**b)** Relationship between TauL1-seedling PC (*x*) and habitat soil available water capacity (*y*). (**c**) Relationship between soil available water capacity (*x*) and electrical conductivity (*y*). Eight accessions are not plotted due to missing values. In all panels, “H”, “L”, and “@” denote the HighQ2, LowQ2, and Q3 accessions, respectively. For each accession, the seedling phenotype is colored according to the key in (**a**).

The natural variation patterns found under the NaCl conditions showed that seedling growth ability was larger in the HighQ2 accessions than in the LowQ2 and Q3 accessions. However, the ecological functions of this larger seedling growth ability were not clear and, as so, the TauL1b accessions’ seedling phenotypes association with natural habitat soil salinity and available water capacity was assessed. Of the 72 TauL1b accessions for which TauL1-seedling PC values were obtained, 41 displayed the long first-leaf phenotypes, whereas 31 displayed the short first-leaf phenotypes (Supplementary Table S6). Of these, the habitat of 35 long first-leaf and 29 short first-leaf accessions had usable soil salinity estimates. Available water capacity estimates were obtained for 41 long first-leaf and 31 short first-leaf accessions. Soil salinity estimates showed notable patterns: 13 out of 35 long first-leaf accessions (37.1%) were sampled in increased salinity soil habitats (electrical conductivity values > 1 dS/m), whereas only two out of 29 short first-leaf accessions (6.9%) were sampled in such habitats (Fig. 5a). The patterns found for the available water capacity estimates were less obvious, but the proportion of accessions sampled in reduced available water capacity soil habitats (<15 cm/m) was higher in long first-leaf accessions (28 out of 41, 68.3%) than in short first-leaf accessions (13 out of 31, 41.9%) (Fig. 5b). A stepwise multiple regression that used the 64 accessions having no missing values selected electrical conductivity, but not available water capacity and the interaction, as the independent predictor of TauL1-seedling PC. A simple linear regression showed that electrical conductivity had significant effect on the TauL1-seedling PC (percentage of explained variance *R*^2^ = 0.13, *F*-value = 9.49, parameter estimate = 0.62, *P* = 0.00).

The edaphic conditions of the habitats were classified into three categories: (1) increased salinity (electrical conductivity values > 1 dS/m) and reduced available water capacity (<15 cm/m) (IR); (2) reduced salinity (electrical conductivity values ≤ 1 dS/m) and reduced available water capacity (<15 cm/m) (RR); and (3) reduced salinity (electrical conductivity values ≤ 1 dS/m) and increased available water capacity (≥15 cm/m) (RI). Examination of the distribution of the seedling phenotypes among these categories revealed that 28 out of 35 long first-leaf accessions (80.0%) were sampled in the relatively stressful habitats, i.e., IR and RR soil habitats (Fig. 5c; Supplementary Table S7). Almost all long first-leaf accessions sampled from IR habitats were belonged to HighQ2. Samples collected in RR habitats had similar numbers of long first-leaf (15) and short first-leaf (11) accessions, whereas most accessions sampled from RI habitats had the short first-leaf phenotype (16/23, 69.6%). Thus, the distribution of long first-leaf and short first-leaf accessions was significantly different between habitats differing in edaphic conditions (Fisher’s exact test, *P* = 0.00). These findings indicated that increased seedling growth ability under NaCl-induced-stress conditions likely had an ecological role in TauL1b’s adaptation to natural edaphic stress.

Furthermore, we found that the long first-leaf TauL1b accessions sampled in the IR and RR soil habitats had discontinuous north-south genetic structure, whereas their phenotypic variation had no obvious geographic patterns (Supplementary Table S7; Supplementary Fig. S4). Similar numbers of HighQ2 and LowQ2 accessions were sampled in RR soil habitats (nine and six, respectively) (Supplementary Table S7). The nine HighQ2 accessions had generally high Q2 values (>0.8) (i.e., the proportion of alleles inferred to have originated from one of the two large TauL1b genepools) and all but one occurred in the south, whereas six LowQ2 accessions had low Q2 values (<0.05) and occurred in the north (Supplementary Fig. S4). The TauL1-seedling PC values of these accessions varied from 0.25 to 3.95 (mean = 1.35) in the north (latitude ≥ 35°) and from 0.12 to 3.00 (mean = 1.25) in the south (latitude < 35°). Among the 13 long first-leaf accessions sampled in IR soil habitats, 12 belonged to HighQ2 and their genetic variation was geographically structured: Q2 values of the southern accessions were usually higher than those of the northern accessions (Supplementary Fig. S4). The TauL1-seedling PC values of these accessions ranged from 1.06 to 2.85 (mean = 1.69) in the north (latitude ≥ 35°) and from 0.63 to 2.58 (mean = 1.48) in the south (latitude < 35°).

### Impact of salt tolerance during seedling growth on species range expansion

**Figure 6:**
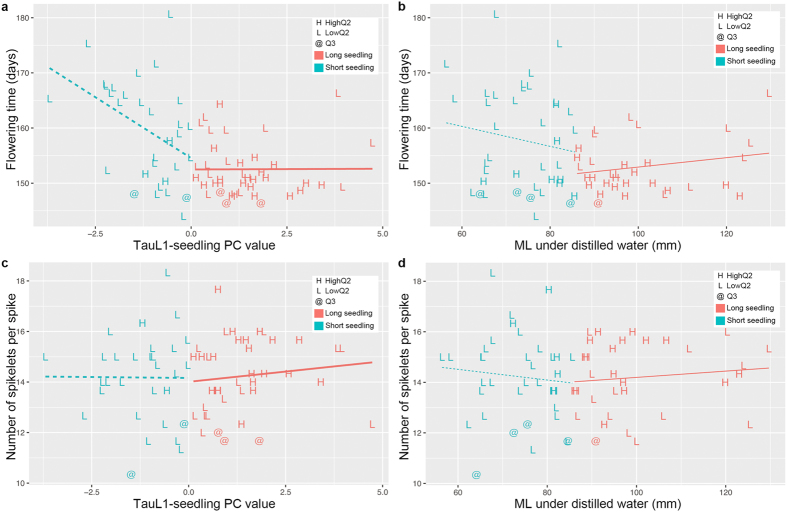
Association of the seedling phenotypes with reproductive traits in 72 TauL1b accessions. (**a**) Relationship between TauL1-seedling PC (*x*) and flowering time (*y*). (**b**) Relationship between ML (distilled water) (*x*) and flowering time (*y*). (**c**) Relationship between TauL1-seedling PC (*x*) and the number of spikelets per spike (*y*). (**d**) Relationship between ML (distilled water) (*x*) and the number of spikelets per spike (*y*). In all panels, “H”, “L”, and “@” denote the HighQ2, LowQ2, and Q3 accessions, respectively. For each accession, the seedling phenotype is colored according to the key. See Supplementary Table S8 for regression analysis details.

Given that NaCl-induced stress tolerance during seedling growth is ecologically functional, adaptation to salt-affected soils might have had an important role in TauL1 accessions’ migration and colonization of central Asian habitats. If so, what impact did salt tolerance have on *Ae. tauschii* eastward range expansion relative to other traits of ecological importance such as flowering time and seed numbers? To address this question, we first examined the association of TauL1b seedling phenotypes with flowering time and the numbers of spikelets per spike. Accessions presenting the long first-leaf phenotype under NaCl-induced-stress conditions mostly showed early flowering, whereas short first-leaf accessions tended to show increased flowering time; this negative association was statistically significant (*P* = 0.02) (Fig. 6a). Interestingly, this negative association was less evident and not significant for the regression performed on MLs under the distilled-water condition (Fig. 6b). These findings suggested that the NaCl-induced-tolerance during seedling growth might be involved with the expression of rapid life cycle in the natural salt-affected soil habitats. No apparent association was found between the numbers of spikelets per spike and the NaCl-stressed and distilled-water seedling phenotypes (Fig. 6c,d).

**Figure 7:**
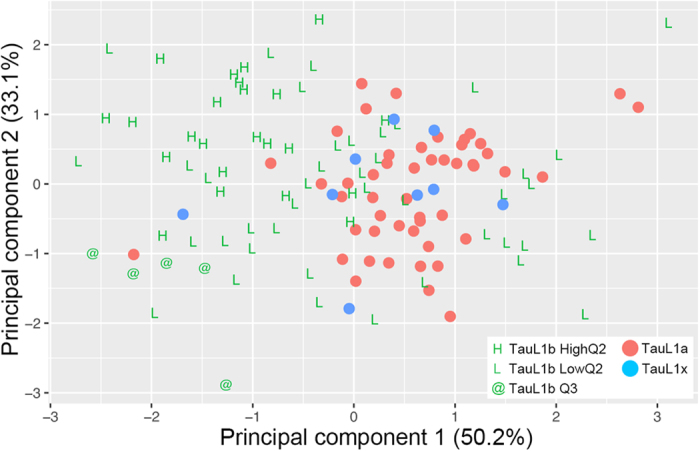
Relationship between TauL1 accessions based on the variation of TauL1-seedling PC and reproductive traits. The genetic group of each accession is indicated according to the key. The first and second principal components (PC1 and PC2) explained 50.2% and 33.1% of the correlation structure, respectively.

Next, we evaluated the impact of NaCl-induced-stress tolerance during seedling growth through a PCA of the TauL1 accessions. In this PCA, the among-accession correlation matrix was used. This allowed obtaining the distribution of TauL1a, TauL1b, and TauL1x accessions in a multidimensional space defined by the rectangular coordinate axes of TauL1-seedling PC, flowering time, and the number of spikelets per spike. This PCA considered only the 127 accessions that had no missing values for these traits. The first and second principal components (PC1 and PC2) explained 50.2% and 33.1% of the correlation structure, respectively. The eigenvectors of PC1 were −0.63 (TauL1-seedling PC, PC loading = −0.77), 0.70 (flowering time, PC loading = 0.86), and 0.33 (number of spikelets per spike, PC loading = 0.40), whereas the eigenvectors of PC2 were 0.45 (TauL1-seedling PC, PC loading = 0.45), −0.01 (flowering time, PC loading = −0.01), and 0.89 (number of spikelets per spike, PC loading = 0.89). The plot of the 127 accessions in the space defined by PC1 and PC2 showed that the TauL1b accessions, particularly the HighQ2 and Q3 accessions, had generally smaller PC1 values than other accessions (Fig. 7). The eigenvectors of PC1 indicated that TauL1b accessions had characteristically higher under-stress seedling growth ability, earlier flowering, and larger per-spike spikelet numbers than TauL1a and TauL1x accessions. Comparable absolute PC loadings were found for TauL1-seedling PC (0.77) and flowering time (0.86), and both loadings were larger than that found for the number of spikelets per spike (0.40). These results suggested that salt tolerance during seedling growth and flowering time might have been important factors for *Ae. tauschii*’s eastward range expansion.

## Discussion

In this study, the habitat soil property estimates were taken from the 30 by 30 arcsec resolution soil datasets. The electrical conductivity and available water capacity values were not validated using soil samples where the plants actually grow. Therefore, the use of these soil datasets may have inherent limitations on analyses in terms of accuracy. Nevertheless, results pointed out a probable association between natural variations in the patterns of *Ae. tauschii* NaCl-induced-stress tolerances and local edaphic variables. Thus, this work highlighted the potential of these soil datasets for use in the ecogeographic studies of *Ae. tauschii*.

Results also suggested that stress tolerances might indeed have ecological functions that are involved in plant’s adaptation to salt-affected soils. Lineages’ pairwise comparisons revealed the higher germinability and seedling growth ability of the geographically widespread TauL1 under stress conditions in relation to other lineages. Seedling growth ability of TauL1b accessions, which were responsible for the eastward range expansion of the TauL1 lineage, was higher than that of TauL1a accessions under control and NaCl-induced-stress conditions, whereas the germinability of both sublineages was similar. The distinct effects of NaCl-induced stress on germination and seedling growth ability are consistent with the genetic evidence that different quantitative trait loci control these two traits in wheat^33^. Therefore, the increased seedling growth ability of TauL1b might be one of the key phenotypes that facilitated the colonization of the semi-arid salt-affected central Asian habitats. Both genetic and geographic factors influenced the variation patterns of TauL1b seedling growth ability under stress (Supplementary Table S6). Because long first-leaf accessions mostly occurred in the southern part of this lineage distribution range, their increased seedling growth ability might have been involved in the adaptation to the edaphic conditions of the southern habitats (Fig. 4).

The electrical conductivity estimates of the *Ae. tauschii* habitats are lower than the soil salinity (electrical conductivity) threshold of yield decline for common wheat (6.0 dS/m)^34^. The threshold of yield decline is not known for *Ae. tauschii*, but one obvious question here would be whether the habitats’ edaphic conditions actually had an impact on the distribution of *Ae. tauschii*, given such low electrical conductivity estimates. In general, low salinity levels can exert physiological stress on plants. Furthermore, plant responses to salinity vary with the developmental stage at time of exposure^10^. Therefore, the findings of this study may suggest that *Ae. tauschii* plants are relatively sensitive to soil salts during early developmental stages under natural conditions. The fact that common wheat is less salt-tolerant during emergence and seedling stage is consistent with this view^34^.

Each TauL1b genetic group contained long first-leaf accessions (Supplementary Table S6). In addition, the genetic variations of the long first-leaf accessions had clear patterns of differentiation and geographic structure (Supplementary Table S5; Supplementary Fig. S4). Given that *Ae. tauschii* is a selfing species, therefore reproducing in a scenario of reduced gene flow, one possible explanation for these observations would be that, in *Ae. tauschii*, NaCl-induced-stress tolerance evolved through multiple pathways of adaptation to the natural edaphic stresses encountered during the species range expansion. For example, an increased capacity of water uptake might be a mechanism responsible for TauL1b’s increased seedling growth ability. In fact, the TauL1b accessions growing in semi-arid habitats also had longer MLs than the TauL1a and TauL1x accessions in control conditions (Supplementary Fig. S3), suggesting that tolerance to osmotic stress was a critical factor in the species’ range expansion toward east. Furthermore, ionic stress tolerance might be involved in TauL1b’s increased seedling growth ability under stress: salinity stress might be more intense in IR than in RR habitats, but the proportion of long first-leaf accessions was not lower in IR habitats (13/15, 86.7%) than in RR habitats (15/26, 57.7%) (Fisher’s Exact Test, *P* = 0.08) (Supplementary Table S7). It is not clear why there were no obvious geographic patterns in the phenotypic variations of the long first-leaf TauL1b accessions (Supplementary Table S7; Supplementary Fig. S4). Use of high resolution soil datasets might solve this issue. Refined experimental approaches taking into account complex phenotypic effects, such as transgenerational plasticity, would enable to better assess the degree of salt tolerance providing suitable phenotype data for inferring the genes controlling seedling growth ability under natural salt-stress conditions^35^. Detailed knowledge on the genetic mechanism(s) underlying the evolution of seedling growth ability would contribute to better understanding of the adaptation of *Ae. tauschii* to salt-affected soils during its eastward range expansion.

*Ae. tauschii*’s life history and phenological traits, particularly seed production and flowering time, are thought to have had a role in this species dispersal and adaptation in central Asia^19^. The PCA performed using seedling growth ability, flowering time, and spikelet number per-spike datasets provided information on traits/phenotypes contributions to *Ae. tauschii*’s eastward range expansion. The influence of seedling growth ability under stress was large and similar to that found for flowering time, suggesting that adaptive phenotypic changes in physiological traits might have been important factors in the eastward range expansion of *Ae. tauschii*. Because seedling growth traits are expressed early in development, they are usually exposed to natural selection pressures before other traits are expressed. For this reason, early seedling growth can have a particular adaptive importance under natural conditions. Because the seedling growth ability was assessed using individuals undergoing early post-germination stages, the influence its performance under stress conditions had on the eastward range expansion of *Ae. tauschii* might be consistent with the influence that has been attributed to germination behavior in determining species ecological and geographic ranges^36^. We note that the observed association between seedling growth ability under stress and early flowering in *Ae. tauschii* parallels a case in *Medicago* in which flowering time was shown to play a major role in adaptation to saline soils^9^. *Ae. tauschii*’s eastward range expansion was probably a complex evolutionary process that involved adaptive changes in multiple ecologically functional traits, including phenological, life history, and physiological traits.

Given that *Ae. tauschii* is one of the progenitors of common wheat^11^,^12^, a widely accepted scenario for its evolution is that in which male *Ae. tauschii* crossed with a cultivated female form of the tetraploid wheat *Triticum turgidum* L. under natural conditions. This cross gave rise to triploid F_1_ hybrids, which, in turn, produced F_2_ hybrids, the direct ancestors of current common wheat, through natural genome duplication via union of male and female unreduced 2n gametes^37^. Previous work indicated that the *T. turgidum* - *Ae. tauschii* natural hybridization involved a few *Ae. tauschii* populations that were genetically close to the TauL2 lineage^17^. This means that an extensive portion of *Ae. tauschii* genetic variation has literally been left in the wild. Furthermore, the *Ae. tauschii* germplasm can be readily incorporated into the common wheat germplasm using synthetic common wheats that can be produced by artificially crossing *T. turgidum* with *Ae. tauschii*^38^. Hence, the *Ae. tauschii* germplasm represents one of the most important genetic resources for common wheat breeding.

Several studies regarding *Ae. tauschii*’s salt tolerance characterization and use in common wheat breeding programs have been performed^39^,^40^,^41^,^42^,^43^. In wheat, salt tolerance is a rather complex trait associated with the ability to maintain low Na^+^ and high K^+^ concentrations, which is accomplished by limiting the concentration of Na^+^ that enters the xylem^44^,^45^,^46^,^47^. *Ae. tauschii* contributed a locus (*Kna1*) that controls the concentration of Na^+^ entering the xylem to bread wheat (a form of common wheat) through a natural hybridization event. Therefore, it is a great source of natural genetic variation for controlling Na^+^ accumulation in leaves^43^,^48^,^49^. In contrast, little was known about how salt stress affects *Ae. tauschii*’s germination behavior and early seedling growth.

Results of the present study suggest that TauL1b accessions may be useful for salt-tolerant wheat breeds, because, in general, uniform and rapid seedling growth are traits of great agronomic value. TauL1b accessions also have phenological (early flowering) and reproductive (high seed productivity) traits suitable for wheat production, and may serve as a pool of novel alleles for controlling these traits, as the TauL1b genepool is relatively distantly related to the D-genome of common wheat (Supplementary Fig. S1). Further studies are needed to provide practical knowledge on the ecology and genetics of *Ae. tauschii*’s germination and seedling salt tolerances.

## Additional Information

**How to cite this article**: Saisho, D. *et al*. Salt tolerance during germination and seedling growth of wild wheat *Aegilops tauschii* and its impact on the species range expansion. *Sci. Rep.* **6**, 38554; doi: 10.1038/srep38554 (2016).

**Publisher’s note:** Springer Nature remains neutral with regard to jurisdictional claims in published maps and institutional affiliations.

## Supplementary Material

Supplementary Information
