# Supplementary material for: Salt tolerance during germination and seedling growth of wild wheat Aegilops tauschii and its impact on the species range expansion
Source: Sci Rep. 2016 Dec 8;6:38554. doi: 10.1038/srep38554 (PMC5143976; doi:10.1038/srep38554)
Supplement: Supplementary Information [file srep38554-s1.pdf]

Salt tolerance during germination and seedling growth of wild wheat *Aegilops tauschii* and its impact on the species range expansion

Daisuke Saisho, Shigeo Takumi, Yoshihiro Matsuoka

Supplementary Information

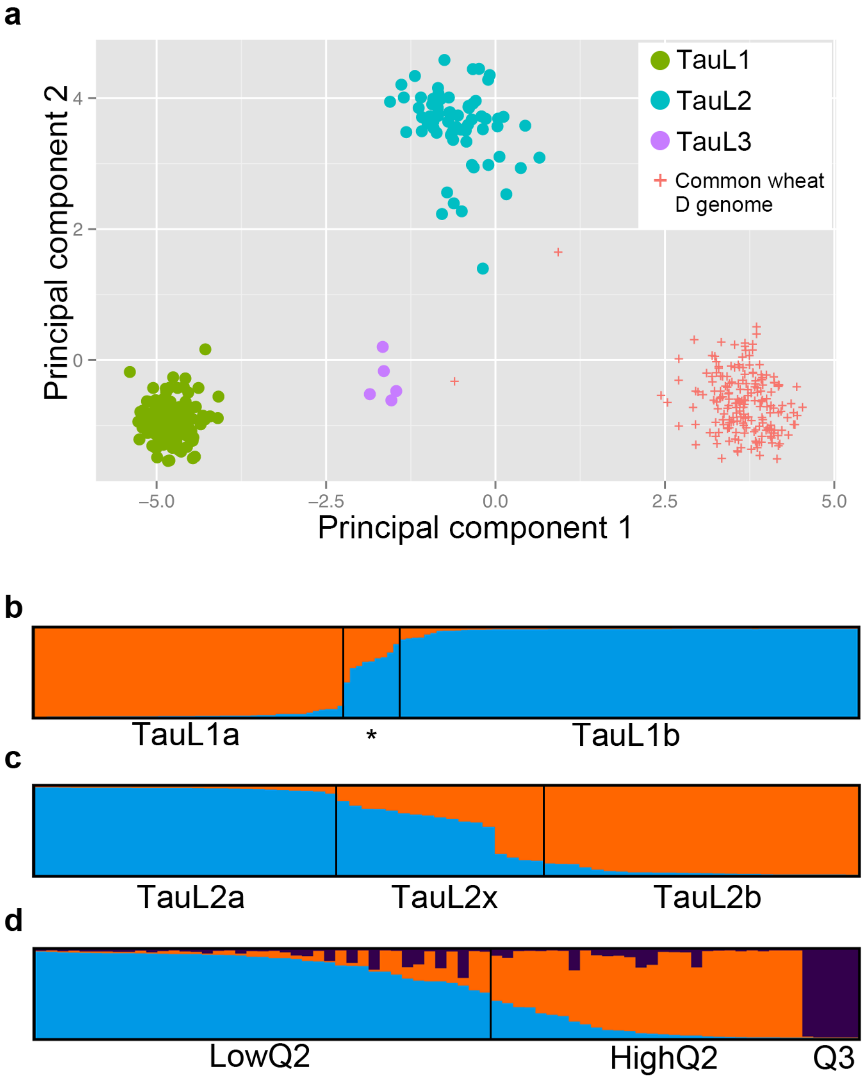

Figure S1. Intraspecific lineages, sublineages, and groups of *Ae. tauschii*.

a. Principal component analysis plot based on molecular marker variations showing the relationships between *Ae. tauschii* lineages and the common wheat D-genome. The first component (*x*) accounts for 41.8% and the second (*y*) for 8.5% of the total variance. Redrawn from Matsuoka, Y. *et al*. Genetic basis for spontaneous hybrid genome doubling during allopolyploid speciation of common wheat shown by natural variation analyses of the paternal species. PLoS One **8**, e68310; DOI: 10.1371/journal.pone.0068310 (2013).

b. Proportional membership plot of 133 TauL1 accessions based on the STRUCTURE analysis of the polymorphisms at 160 molecular marker loci showing the TauL1a and TauL1b sublineages. The asterisk denotes TauL1x, a group of accessions genetically intermediate to TauL1a and TauL1b. Taken from Matsuoka, Y., Takumi, S. & Kawahara, T. Intraspecific lineage divergence and its association with reproductive trait change during species range expansion in central Eurasian wild wheat *Aegilops tauschii* Coss. (Poaceae). BMC Evol. Biol. **15**, 213; DOI: 10.1186/s12862-015-0496-9 (2015).

c. Proportional membership plot of 68 TauL2 accessions based on the STRUCTURE analysis of the polymorphisms at 237 molecular marker loci showing the TauL2a and TauL2b sublineages.TauL2x is a group of accessions genetically intermediate to TauL2a and TauL2b. Taken from Matsuoka, Y., Takumi, S. & Kawahara, T. Intraspecific lineage divergence and its association with reproductive trait change during species range expansion in central Eurasian wild wheat *Aegilops tauschii* Coss. (Poaceae). BMC Evol. Biol. **15**, 213; DOI: 10.1186/s12862-015-0496-9 (2015).

d. Proportional membership plot of 74 TauL1b accessions based on the STRUCTURE analysis of the polymorphisms at 118 molecular marker loci showing three genetic groups, LowQ2, HighQ2, and Q3. Taken from Matsuoka, Y., Takumi, S. & Kawahara, T. Intraspecific lineage divergence and its association with reproductive trait change during species range expansion in central Eurasian wild wheat *Aegilops tauschii* Coss. (Poaceae). BMC Evol. Biol. **15**, 213; DOI: 10.1186/s12862-015-0496-9 (2015).

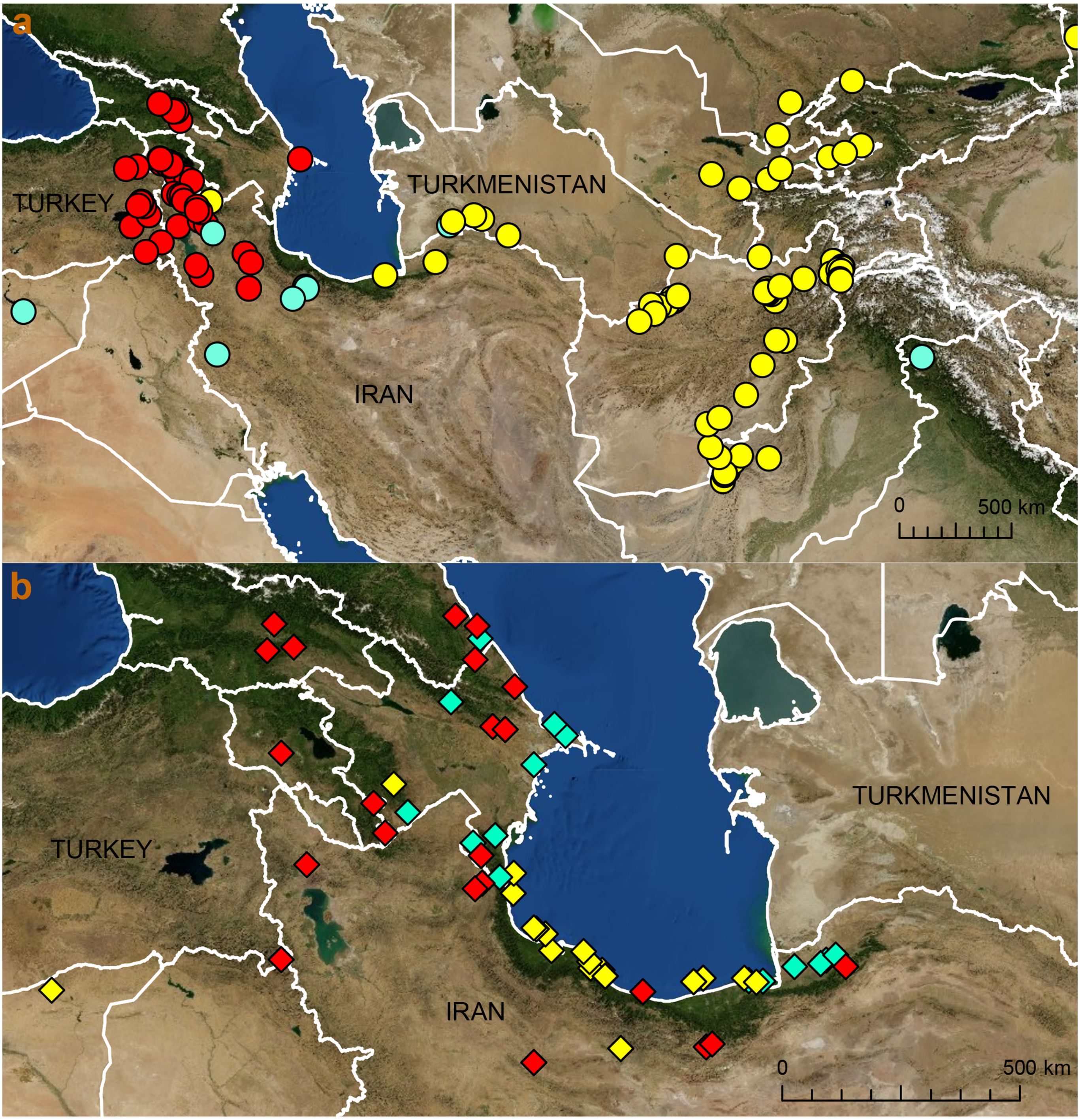

Figure S2. TauL1 (circles) and TauL2 (squares) *Ae. tauschii* accessions' geographic distribution. a. TauL1a (red), TauL1b (yellow), and TauL1x (blue) accessions. The six TauL1 lineage accessions representing adventive populations in the Shaanxi and Henan provinces are not shown. The map was created using the ArcGIS for Desktop software (ver. 10.2.2) [ESRI, Redlands, CA, USA, ArcGIS for Desktop., (2014) http://www.esrij.com/products/arcgis-for-desktop/ (Date of access:25/10/2016)] and the World Imagery basemap layer [esri,World Imagery., (2009) https://www.arcgis.com/home/item.html?id=10df2279f9684e4a9f6a7f08febac2a9 (Date of access:25/10/2016)]. Sources: Esri, DigitalGlobe, Earthstar Geographics, CNES/Airbus DS, GeoEye, USDA FSA, USGS, Getmapping, Aerogrid, IGN, IGP, and the GIS User Community.

b. TauL2a (red), TauL2b (yellow), TauL2x (blue) accessions. The TauL3 accessions, endemic to Georgia, are not shown. The map was created using the ArcGIS for Desktop software (ver. 10.2.2) [ESRI, Redlands, CA, USA, ArcGIS for Desktop., (2014) http://www.esrij.com/products/arcgis-for-desktop/ (Date of access:25/10/2016)] and the World Imagery basemap layer [esri,World Imagery., (2009) https://www.arcgis.com/home/item.html?id=10df2279f9684e4a9f6a7f08febac2a9 (Date of access:25/10/2016)]. Sources: Esri, DigitalGlobe, Earthstar Geographics, CNES/Airbus DS, GeoEye, USDA FSA, USGS, Getmapping, Aerogrid, IGN, IGP, and the GIS User Community.

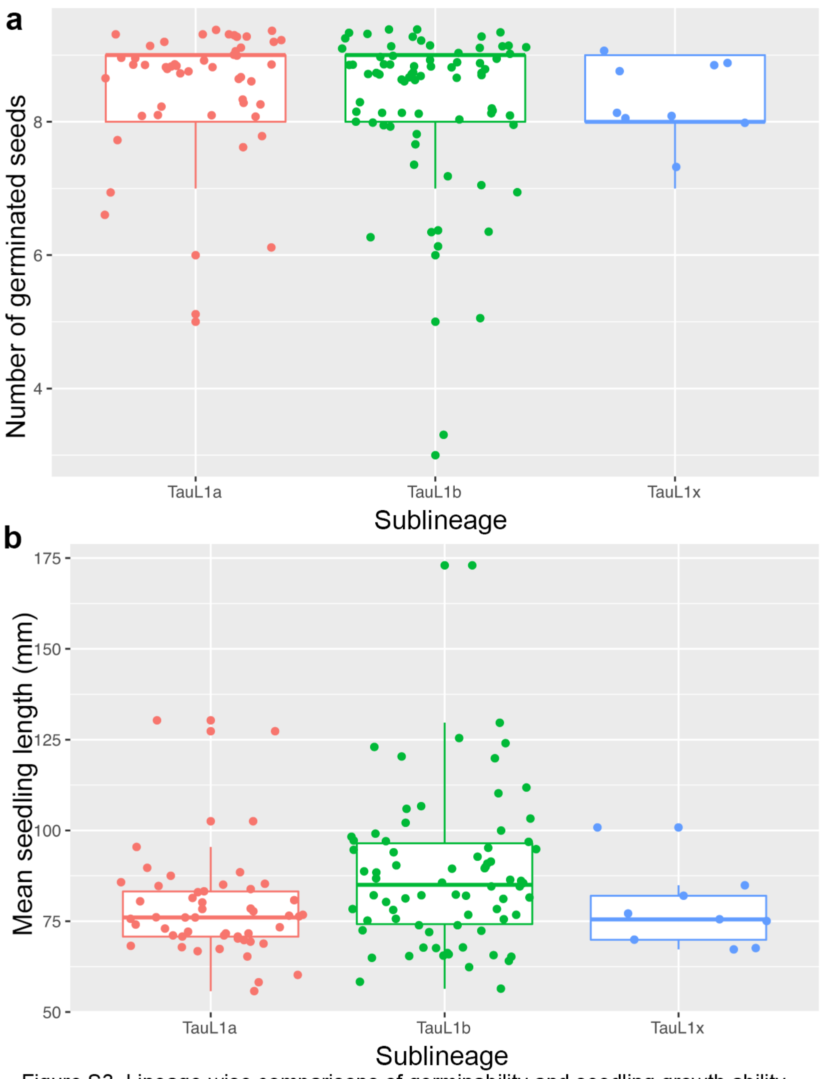

Figure S3. Lineage-wise comparisons of germinability and seedling growth ability in control (distilled water) condistions. a. Box and dot plots of the number of germinated seeds. b. Box and dot plots of the mean first leaf lengths.

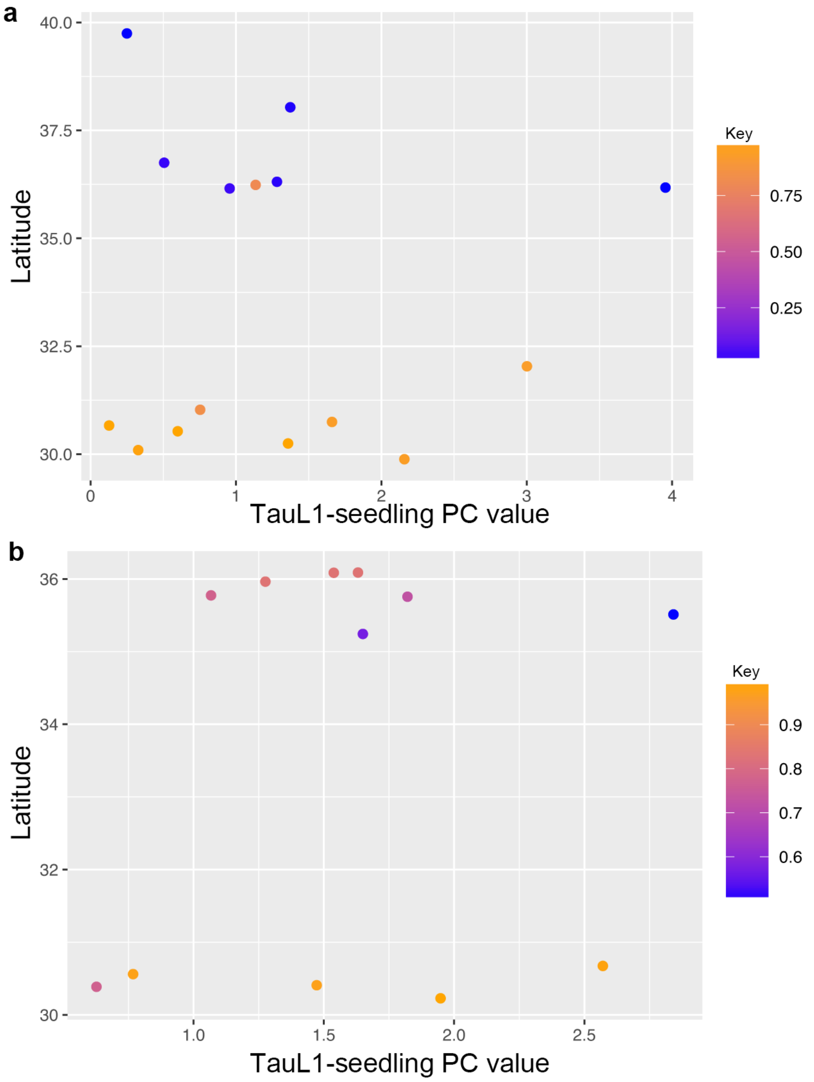

Figure S4. Population structure of the long-seedling TauL1b accessions sampled in habitats with different habitat soil conditions. a. Relationship between TauL1-seedling PC (*x*) and latitude (*y*) of the accessions sampled in the RR habitats. b. Relationship beween TauL1-seedling PC (*x*) and latitude (*y*) of the accessions sampled in the IR habitats. In each panel, the gradient color key indicates the Q2 value of each accession.

Table S1 The *Ae. tauschii* accessions used and their genetic and phenotypic properties. Source codes are IPK for Institut für Pflanzengenetik und Kulturpflanzenforschung, CGN for Centre for Genetic Resources, The Netherlands, ICARDA for International Center for Agricultural Research in the Dry Areas, KYOTO for Plant Germ-plasm Institute of Kyoto University, NBRP for National BioResources Project, OKAYAMA for Dr. Kenji Kato, Okayama University, and USDA for US Department of Agriculture. Hyphens indicate that the value is not available.

No.	Accession	Origin	Latitude	Longitude	Habitat soil salinity (Electrical Conductivity, dS/m)	Habitat soil available water capacity (cm/m)	Sublineage/group	TauL1b gentic group	TauL1-germination PC value	TauL1-seedling PC value	Source
1	CGN 10734	-	-	-	-	-	TauL1a	-	-0.02	0.65	CGN
2	IG 126273	Armenia	40	44.96	0.10	19	TauL1a	-	-1.56	-0.44	ICARDA
3	IG 126280	Armenia	39.9	44.94	0.10	19	TauL1a	-	1.74	-0.47	ICARDA
4	IG 126293	Armenia	39.8	45.33	0.10	19	TauL1a	-	-1.14	1.60	ICARDA
5	IG 126353	Armenia	39.71	45.57	0.10	19	TauL1a	-	4.01	-0.43	ICARDA
6	IG 47196	Azerbaijan	40.38	49.88	0.10	15	TauL1a	-	-0.84	-0.98	ICARDA
7	IG 48747	Armenia	40.28	44.63	0.10	19	TauL1a	-	-2.20	-1.17	ICARDA
8	IG 48748	Armenia	40.18	44.67	0.10	19	TauL1a	-	-2.84	-0.60	ICARDA
9	IG 48758	Armenia	40.25	44.33	0.10	19	TauL1a	-	1.65	-0.87	ICARDA
10	IG 49095	Iran	36.33	47.83	0.10	18	TauL1a	-	0.07	0.48	ICARDA
11	AE 933	Georgia	41.85	44.79	0.10	13	TauL1a	-	4.01	-1.67	IPK
12	KU-2113	Iran	36.76	45.94	0.50	21	TauL1a	-	-1.84	-1.36	KYOTO/NBRP

13	KU-2115	Iran	37.07	45.74	0.10	23	TauL1a	-	0.98	-0.92	KYOTO/NBRP
14	KU-2116	Iran	38.29	45.02	0.70	20	TauL1a	-	1.04	0.24	KYOTO/NBRP
15	KU-2120	Iran	38.49	45.88	0.10	18	TauL1a	-	2.19	0.06	KYOTO/NBRP
16	KU-2121	Iran	38.38	46.13	0.10	13	TauL1a	-	-1.17	-0.48	KYOTO/NBRP
17	KU-2131	Turkey	38.29	43.15	1.10	12	TauL1a	-	1.19	0.30	KYOTO/NBRP
18	KU-2132	Turkey	38.29	43.15	1.10	12	TauL1a	-	-0.93	-0.05	KYOTO/NBRP
19	KU-2133	Turkey	38.64	43.83	1.10	12	TauL1a	-	2.80	1.17	KYOTO/NBRP
20	KU-2136	Turkey	38.92	43.62	1.10	12	TauL1a	-	-2.29	-0.04	KYOTO/NBRP
21	KU-2137	Turkey	39.07	43.54	1.10	12	TauL1a	-	1.74	0.57	KYOTO/NBRP
22	KU-2138	Turkey	39.04	43.51	1.10	12	TauL1a	-	1.04	-0.28	KYOTO/NBRP
23	KU-2140	Turkey	39.04	43.51	1.10	12	TauL1a	-	0.83	-0.33	KYOTO/NBRP
24	KU-2141	Turkey	38.94	43.41	1.10	12	TauL1a	-	-0.38	0.40	KYOTO/NBRP
25	KU-2142	Iran	39.32	44.84	0.10	18	TauL1a	-	1.17	-0.33	KYOTO/NBRP
26	KU-2143	Iran	39.26	45.07	0.50	21	TauL1a	-	0.04	-0.32	KYOTO/NBRP
27	KU-2144	Iran	39.24	45.16	0.30	13	TauL1a	-	-3.53	-0.58	KYOTO/NBRP
28	KU-2145	Iran	39.1	45.24	0.50	21	TauL1a	-	-0.99	-2.16	KYOTO/NBRP
29	KU-2148	Iran	38.75	45.9	0.10	18	TauL1a	-	-3.02	0.29	KYOTO/NBRP
30	KU-2149	Iran	38.9	45.76	0.50	21	TauL1a	-	-0.41	0.55	KYOTO/NBRP
31	KU-2150	Iran	38.8	45.77	0.50	21	TauL1a	-	-1.80	2.09	KYOTO/NBRP
32	KU-2151	Iran	37.44	47.67	0.10	18	TauL1a	-	0.68	-0.07	KYOTO/NBRP
33	KU-2152	Iran	37.15	47.93	0.10	13	TauL1a	-	2.16	-0.79	KYOTO/NBRP

34	KU-2809	Armenia	40.25	44.62	0.10	19	TauL1a	-	3.92	-0.15	KYOTO/NBRP
35	KU-2810	Armenia	40.25	44.62	0.10	19	TauL1a	-	2.50	-0.32	KYOTO/NBRP
36	KU-2814	Armenia	40.25	44.62	0.10	19	TauL1a	-	1.80	-1.94	KYOTO/NBRP
37	KU-2816	Armenia	40.22	44.55	0.10	19	TauL1a	-	2.50	-0.95	KYOTO/NBRP
38	KU-2821	Armenia	40.2	44.75	0.10	19	TauL1a	-	-1.44	-1.22	KYOTO/NBRP
39	KU-2822A	Armenia	40.39	44.27	0.20	14	TauL1a	-	2.04	-0.29	KYOTO/NBRP
40	KU-2823	Armenia	40.38	44.3	0.20	14	TauL1a	-	1.28	-0.63	KYOTO/NBRP
41	KU-2824	Armenia	40.38	44.3	0.20	14	TauL1a	-	5.20	-0.12	KYOTO/NBRP
42	KU-2826	Georgia	41.55	45.1	0.10	13	TauL1a	-	-2.47	-1.78	KYOTO/NBRP
43	KU-2828	Georgia	41.84	44.94	0.10	18	TauL1a	-	-1.93	-1.53	KYOTO/NBRP
44	KU-2834	Georgia	41.82	44.82	0.10	13	TauL1a	-	-2.84	-2.38	KYOTO/NBRP
45	KU-2836	Georgia	42.07	44.26	0.10	19	TauL1a	-	-1.93	0.14	KYOTO/NBRP
46	AT 47	China	34.78	109.25	0.40	16	TauL1a	-	-4.74	1.15	OKAYAMA
47	PI 486270	Turkey	37.78	44.33	0.10	13	TauL1a	-	2.41	0.15	USDA
48	PI 486274	Turkey	40.15	43.37	1.60	18	TauL1a	-	-2.08	-0.22	USDA
49	PI 486277	Turkey	40.08	42.93	1.60	18	TauL1a	-	1.04	-0.01	USDA
50	PI 554319	Turkey	37.48	43.72	0.10	13	TauL1a	-	2.04	-1.24	USDA
51	CGN 10767	Pakistan	30.42	66.97	2.40	13	TauL1b	HighQ2	-3.05	1.47	CGN
52	CGN 10768	Pakistan	30.38	67	2.40	13	TauL1b	HighQ2	1.65	0.63	CGN
53	CGN 10769	Pakistan	30.55	66.88	2.40	13	TauL1b	HighQ2	1.80	0.75	CGN
54	CGN 10770	Pakistan	30.25	67.03	0.10	13	TauL1b	HighQ2	-2.77	1.36	CGN

55	CGN 10771	Pakistan	30.22	67.01	2.40	13	TauL1b	HighQ2	0.68	1.94	CGN
56	IG 108561	Pakistan	29.88	66.83	0.10	13	TauL1b	HighQ2	1.19	2.16	ICARDA
57	IG 120736	Uzbekistan	39.92	66.37	0.10	18	TauL1b	HighQ2	1.65	-1.19	ICARDA
58	IG 46663	Pakistan	30.53	67.25	0.10	13	TauL1b	HighQ2	-3.05	0.60	ICARDA
59	IG 46666	Pakistan	30.75	67.55	0.10	13	TauL1b	HighQ2	4.53	1.66	ICARDA
60	IG 46682	Pakistan	30.67	68.67	0.10	13	TauL1b	HighQ2	0.37	0.12	ICARDA
61	IG 48565	Uzbekistan	40.45	71.07	0.30	18	TauL1b	HighQ2	-1.62	-0.56	ICARDA
62	KU-20-6	Pakistan	30.08	66.9	0.10	13	TauL1b	HighQ2	-2.35	0.33	KYOTO/NBRP
63	KU-2001	Pakistan	30.15	66.9	0.10	13	TauL1b	HighQ2	-8.16	-	KYOTO/NBRP
64	KU-2003	Pakistan	30.15	66.9	0.10	13	TauL1b	HighQ2	-5.99	-	KYOTO/NBRP
65	KU-2006	Pakistan	30.69	66.67	2.40	13	TauL1b	HighQ2	-1.99	2.58	KYOTO/NBRP
66	KU-2008	Pakistan	31.03	66.33	0.10	13	TauL1b	HighQ2	-1.75	0.76	KYOTO/NBRP
67	KU-2010	Afghanistan	31.83	66.21	-	18	TauL1b	HighQ2	-2.04	3.41	KYOTO/NBRP
68	KU-2012	Afghanistan	32.03	66.69	0.10	13	TauL1b	HighQ2	-1.69	3.00	KYOTO/NBRP
69	KU-2039	Afghanistan	36.24	68.59	1.00	13	TauL1b	HighQ2	-1.23	1.13	KYOTO/NBRP
70	KU-2043	Afghanistan	37.35	64.98	0.00	6	TauL1b	HighQ2	1.80	-0.67	KYOTO/NBRP
71	KU-2044	Afghanistan	36.08	65.03	2.40	13	TauL1b	HighQ2	-2.20	1.62	KYOTO/NBRP
72	KU-2056	Afghanistan	35.95	64.9	2.40	13	TauL1b	HighQ2	-0.08	1.28	KYOTO/NBRP
73	KU-2059	Afghanistan	35.74	64.27	2.40	13	TauL1b	HighQ2	4.77	1.83	KYOTO/NBRP
74	KU-2061	Afghanistan	35.78	63.95	2.40	13	TauL1b	HighQ2	-1.56	1.06	KYOTO/NBRP
75	KU-2063	Afghanistan	35.51	64.09	2.40	13	TauL1b	HighQ2	2.16	2.85	KYOTO/NBRP

76	KU-2066	Afghanistan	35.25	63.46	2.40	13	TauL1b	HighQ2	1.28	1.65	KYOTO/NBRP
77	KU-2612	Afghanistan	34.64	68.96	-	18	TauL1b	HighQ2	-0.20	0.75	KYOTO/NBRP
78	PI 476874	Afghanistan	36.08	65.03	2.40	13	TauL1b	HighQ2	-2.04	1.55	USDA
79	IG 120735	Turkmenistan	38.53	57.17	0.30	9	TauL1b	LowQ2	0.98	-1.30	ICARDA
80	IG 123910	Uzbekistan	40.8	72.37	0.30	18	TauL1b	LowQ2	2.08	-0.32	ICARDA
81	IG 126387	Turkmenistan	38.64	56.86	0.10	13	TauL1b	LowQ2	-0.56	-0.29	ICARDA
82	IG 126489	Turkmenistan	38.02	58.24	0.30	9	TauL1b	LowQ2	-1.08	1.37	ICARDA
83	IG 127015	Armenia	39.08	46.31	0.10	19	TauL1b	LowQ2	-4.17	-2.69	ICARDA
84	IG 131606	Kyrgyzstan	42.72	72.01	0.20	16	TauL1b	LowQ2	4.92	-0.03	ICARDA
85	IG 48518	Turkmenistan	38.45	56	0.30	13	TauL1b	LowQ2	-1.32	-0.94	ICARDA
86	IG 48539	Uzbekistan	41.1	69	0.20	18	TauL1b	LowQ2	0.53	-0.96	ICARDA
87	IG 48554	Tajikistan	39.47	67.5	0.10	18	TauL1b	LowQ2	4.77	0.42	ICARDA
88	IG 48559	Tajikistan	39.75	68.63	0.10	13	TauL1b	LowQ2	1.13	0.25	ICARDA
89	IG 48564	Tajikistan	40.08	69.12	0.30	18	TauL1b	LowQ2	1.50	0.16	ICARDA
90	IG 48567	Uzbekistan	40.57	71.7	0.20	18	TauL1b	LowQ2	1.50	-1.04	ICARDA
91	AE 1038	Tajikistan	37.34	68.3	0.30	18	TauL1b	LowQ2	4.77	-0.20	IPK
92	AE 1090	Kazakhstan	42.1	69.53	0.30	12	TauL1b	LowQ2	1.74	-0.38	IPK
93	KU-2016	Afghanistan	32.81	67.75	-	18	TauL1b	LowQ2	-2.69	3.83	KYOTO/NBRP
94	KU-2018	Afghanistan	33.8	68.41	0.10	15	TauL1b	LowQ2	-1.38	4.73	KYOTO/NBRP
95	KU-2022	Afghanistan	34.62	69.31	-	18	TauL1b	LowQ2	1.13	0.36	KYOTO/NBRP
96	KU-2025	Afghanistan	35.91	68.92	-	18	TauL1b	LowQ2	4.92	1.94	KYOTO/NBRP

97	KU-2027	Afghanistan	36.15	68.75		1.00	13	TauL1b	LowQ2	1.44	0.96	KYOTO/NBRP
98	KU-2028	Afghanistan	36.18	68.65		1.00	13	TauL1b	LowQ2	-1.14	3.95	KYOTO/NBRP
99	KU-2032	Afghanistan	36.31	68.6		1.00	13	TauL1b	LowQ2	-2.81	1.28	KYOTO/NBRP
100	KU-2035	Afghanistan	36.22	68.59		1.00	13	TauL1b	LowQ2	-0.47	-2.18	KYOTO/NBRP
101	KU-2042	Afghanistan	36.2	68.52		1.00	13	TauL1b	LowQ2	0.68	-0.81	KYOTO/NBRP
102	KU-2050	Afghanistan	36.01	64.79		2.40	13	TauL1b	LowQ2	-1.23	-0.62	KYOTO/NBRP
103	KU-2051	Afghanistan	35.91	64.88		2.40	13	TauL1b	LowQ2	1.50	-0.89	KYOTO/NBRP
104	KU-2058	Afghanistan	35.81	64.59		2.40	13	TauL1b	LowQ2	-0.08	0.49	KYOTO/NBRP
105	KU-2082	Iran	37.17	55.31		0.10	16	TauL1b	LowQ2	-3.44	0.91	KYOTO/NBRP
106	KU-2087	Iran	36.74	53.29		0.10	14	TauL1b	LowQ2	-3.05	0.51	KYOTO/NBRP
107	KU-2617	Afghanistan	36.64	70.07	-		18	TauL1b	LowQ2	-2.93	-0.02	KYOTO/NBRP
108	KU-2621	Afghanistan	37.19	71.24		0.10	15	TauL1b	LowQ2	-2.20	-2.23	KYOTO/NBRP
109	KU-2624	Afghanistan	36.88	71.39		0.10	15	TauL1b	LowQ2	-3.41	-1.72	KYOTO/NBRP
110	KU-2627	Afghanistan	36.8	71.16	-		18	TauL1b	LowQ2	0.83	-2.09	KYOTO/NBRP
111	KU-2630	Afghanistan	37.02	71.6		0.10	13	TauL1b	LowQ2	0.53	-2.24	KYOTO/NBRP
112	KU-2632	Afghanistan	36.98	71.6		0.10	13	TauL1b	LowQ2	2.65	-1.38	KYOTO/NBRP
113	KU-2633	Afghanistan	36.93	71.45		0.10	15	TauL1b	LowQ2	5.68	-1.86	KYOTO/NBRP
114	KU-2635	Afghanistan	36.88	71.55		0.20	19	TauL1b	LowQ2	-3.57	-0.29	KYOTO/NBRP
115	KU-2636	Afghanistan	36.62	71.5		0.10	15	TauL1b	LowQ2	-1.44	-3.69	KYOTO/NBRP
116	KU-2638	Afghanistan	36.57	71.54		0.10	15	TauL1b	LowQ2	2.50	-2.02	KYOTO/NBRP
117	KU-2639	Afghanistan	36.4	69.11	-		18	TauL1b	LowQ2	-2.90	0.44	KYOTO/NBRP

118	PI 499262	China	44	81	0.30	13	TauL1b	LowQ2		1.65	-0.91	USDA
119	PI 508262	China	44	81	0.30	13	TauL1b	LowQ2		2.65	-0.54	USDA
120	AT 55	China	34.78	109.25	0.40	16	TauL1b	Q3		0.13	1.82	OKAYAMA
121	AT 60	China	34.37	109.25	0.30	18	TauL1b	Q3		-1.32	0.92	OKAYAMA
122	AT 76	China	34.37	107.19	0.40	16	TauL1b	Q3		-2.04	0.76	OKAYAMA
123	AT 80	China	34.78	108.96	0.30	18	TauL1b	Q3		-0.47	-0.12	OKAYAMA
124	PI 508264	China	34	114	0.70	16	TauL1b	Q3		2.04	-1.48	USDA
125	IG 47259	Syria	35.58	38.82	2.90	13	TauL1x	-		0.53	-2.55	ICARDA
126	IG 48042	India	34.08	74.8	0.10	19	TauL1x	-		-1.32	-0.62	ICARDA
127	IG 48508	Turkmenistan	38.33	55.87	0.30	13	TauL1x	-		1.04	-0.47	ICARDA
128	KU-2068	Iran	36.38	50.09	0.10	18	TauL1x	-		-2.14	1.69	KYOTO/NBRP
129	KU-2122	Iran	38.08	46.41	0.10	13	TauL1x	-		-2.81	-0.64	KYOTO/NBRP
130	KU-2153	Iran	36.33	50.15	0.10	18	TauL1x	-		0.19	-0.96	KYOTO/NBRP
131	KU-2154	Iran	35.99	49.61	0.50	12	TauL1x	-		3.16	-0.33	KYOTO/NBRP
132	KU-2157	Iran	34.16	46.56	0.10	13	TauL1x	-		0.44	-1.02	KYOTO/NBRP
133	KU-2619	Afghanistan	37.19	71.27	0.10	15	TauL1x	-		2.65	-1.67	KYOTO/NBRP
134	IG 120863	Dagestan	41.58	48.28	0.10	17	TauL2a	-	-	-		ICARDA
135	IG 126991	Armenia	39.09	46.58	0.10	14	TauL2a	-	-	-		ICARDA
136	IG 47173	Armenia	39.52	46.37	0.20	14	TauL2a	-	-	-		ICARDA
137	IG 47193	Azerbaijan	38.75	48.4	0.10	19	TauL2a	-	-	-		ICARDA
138	IG 47199	Azerbaijan	40.63	48.62	0.10	17	TauL2a	-	-	-		ICARDA

139	IG 47204	Azerbaijan	41.2	49.03	0.10	15	TauL2a	-	-	-	ICARDA
140	IG 48274	Dagestan	42.2	47.92	0.10	17	TauL2a	-	-	-	ICARDA
141	AE 1037	Georgia	41.71	44.35	0.00	18	TauL2a	-	-	-	IPK
142	KU-20-1	Dagestan	42.06	48.33	1.90	18	TauL2a	-	-	-	KYOTO/NBRP
143	KU-20-7	Iran	35.85	51.04	0.10	18	TauL2a	-	-	-	KYOTO/NBRP
144	KU-20-8	Iran	35.87	52.65	0.10	18	TauL2a	-	-	-	KYOTO/NBRP
145	KU-2083	Iran	37.1	55.3	0.10	16	TauL2a	-	-	-	KYOTO/NBRP
146	KU-2086	Iran	35.92	52.77	0.10	13	TauL2a	-	-	-	KYOTO/NBRP
147	KU-2111	Iran	38.35	48.42	0.70	20	TauL2a	-	-	-	KYOTO/NBRP
148	KU-2112	Iran	38.26	48.29	0.50	13	TauL2a	-	-	-	KYOTO/NBRP
149	KU-2118	Iran	38.62	45.1	0.50	21	TauL2a	-	-	-	KYOTO/NBRP
150	KU-2124	Iran	36.72	51.46	0.10	6	TauL2a	-	-	-	KYOTO/NBRP
151	KU-2126	Iran	36.72	51.46	0.10	6	TauL2a	-	-	-	KYOTO/NBRP
152	KU-2155	Iran	35.63	49.4	0.50	13	TauL2a	-	-	-	KYOTO/NBRP
153	KU-2156	Iran	35.63	49.4	0.50	13	TauL2a	-	-	-	KYOTO/NBRP
154	KU-2804	Azerbaijan	40.59	48.85	0.10	17	TauL2a	-	-	-	KYOTO/NBRP
155	KU-2806	Azerbaijan	40.59	48.85	0.10	17	TauL2a	-	-	-	KYOTO/NBRP
156	KU-2811	Armenia	40.25	44.62	0.10	19	TauL2a	-	-	-	KYOTO/NBRP
157	KU-2827	Georgia	41.76	44.85	0.10	13	TauL2a	-	-	-	KYOTO/NBRP
158	KU-2835B	Georgia	42.09	44.48	0.10	18	TauL2a	-	-	-	KYOTO/NBRP
159	PI 486267	Turkey	37.2	44.62	0.10	13	TauL2a	-	-	-	USDA

160	IG 46623	Syria	36.74	40.28	1.00	13	TauL2b	-	-	-	ICARDA
161	IG 47202	Azerbaijan	39.8	46.75	0.10	14	TauL2b	-	-	-	ICARDA
162	KU-20-10	Iran	37.04	50.69	0.10	6	TauL2b	-	-	-	KYOTO/NBRP
163	KU-2069	Iran	35.85	51.04	0.10	18	TauL2b	-	-	-	KYOTO/NBRP
164	KU-2088	Iran	36.92	53.38	0.70	17	TauL2b	-	-	-	KYOTO/NBRP
165	KU-2090	Iran	36.86	53.61	0.70	17	TauL2b	-	-	-	KYOTO/NBRP
166	KU-2091	Iran	36.92	52.6	0.70	17	TauL2b	-	-	-	KYOTO/NBRP
167	KU-2092	Iran	36.92	52.6	0.70	17	TauL2b	-	-	-	KYOTO/NBRP
168	KU-2093	Iran	36.87	52.42	0.70	17	TauL2b	-	-	-	KYOTO/NBRP
169	KU-2096	Iran	36.87	52.42	0.70	17	TauL2b	-	-	-	KYOTO/NBRP
170	KU-2097	Iran	36.87	52.42	0.70	17	TauL2b	-	-	-	KYOTO/NBRP
171	KU-2098	Iran	36.95	50.75	0.10	19	TauL2b	-	-	-	KYOTO/NBRP
172	KU-2100	Iran	37.07	50.47	0.10	19	TauL2b	-	-	-	KYOTO/NBRP
173	KU-2101	Iran	37.17	50.46	0.10	19	TauL2b	-	-	-	KYOTO/NBRP
174	KU-2102	Iran	37.32	50.34	0.10	23	TauL2b	-	-	-	KYOTO/NBRP
175	KU-2103	Iran	37.34	49.73	0.10	23	TauL2b	-	-	-	KYOTO/NBRP
176	KU-2104	Iran	37.59	49.62	0.10	23	TauL2b	-	-	-	KYOTO/NBRP
177	KU-2105	Iran	37.66	49.44	0.10	23	TauL2b	-	-	-	KYOTO/NBRP
178	KU-2106	Iran	37.67	49.4	0.10	23	TauL2b	-	-	-	KYOTO/NBRP
179	KU-2107	Iran	38.19	49.01	0.10	23	TauL2b	-	-	-	KYOTO/NBRP
180	KU-2108	Iran	38.19	49.01	0.10	23	TauL2b	-	-	-	KYOTO/NBRP

181	KU-2109	Iran	38.49	49.02	0.10	13	TauL2b	-	-	-	KYOTO/NBRP
182	KU-2158	Iran	36.95	50.75	0.10	19	TauL2b	-	-	-	KYOTO/NBRP
183	KU-2159	Iran	36.95	50.75	0.10	19	TauL2b	-	-	-	KYOTO/NBRP
184	KU-2160	Iran	36.95	50.75	0.10	19	TauL2b	-	-	-	KYOTO/NBRP
185	IG 120866	Dagestan	41.88	48.38	0.10	17	TauL2x	-	-	-	ICARDA
186	IG 47182	Azerbaijan	39.05	48.67	1.00	19	TauL2x	-	-	-	ICARDA
187	IG 47186	Azerbaijan	40.08	49.4	0.10	15	TauL2x	-	-	-	ICARDA
188	IG 47188	Azerbaijan	40.98	47.83	0.10	18	TauL2x	-	-	-	ICARDA
189	IG 47192	Azerbaijan	38.93	48.25	0.10	19	TauL2x	-	-	-	ICARDA
190	IG 47194	Azerbaijan	40.5	50	0.10	15	TauL2x	-	-	-	ICARDA
191	IG 47203	Azerbaijan	39.38	47.02	0.10	13	TauL2x	-	-	-	ICARDA
192	KU-20-9	Iran	36.88	53.47	0.70	17	TauL2x	-	-	-	KYOTO/NBRP
193	KU-2074	Iran	36.88	53.47	0.70	17	TauL2x	-	-	-	KYOTO/NBRP
194	KU-2075	Iran	36.87	53.73	0.70	17	TauL2x	-	-	-	KYOTO/NBRP
195	KU-2076	Iran	37.1	54.33	5.90	21	TauL2x	-	-	-	KYOTO/NBRP
196	KU-2077	Iran	37.2	54.93	0.50	15	TauL2x	-	-	-	KYOTO/NBRP
197	KU-2078	Iran	37.14	54.83	0.50	15	TauL2x	-	-	-	KYOTO/NBRP
198	KU-2079	Iran	37.14	54.83	0.50	15	TauL2x	-	-	-	KYOTO/NBRP
199	KU-2080	Iran	37.27	55.11	5.90	21	TauL2x	-	-	-	KYOTO/NBRP
200	KU-2110	Iran	38.43	48.76	0.10	19	TauL2x	-	-	-	KYOTO/NBRP
201	KU-2801	Azerbaijan	40.66	49.79	0.10	15	TauL2x	-	-	-	KYOTO/NBRP

202	AE 454	Georgia	41.89	44.8	0.10	18	TauL3	-	-	-	IPK
203	AE 457	Georgia	41.64	44.9	0.10	13	TauL3	-	-	-	IPK
204	AE 929	Georgia	41.89	44.8	0.10	18	TauL3	-	-	-	IPK
205	KU-2829A	Georgia	41.82	44.82	0.10	13	TauL3	-	-	-	KYOTO/NBRP
206	KU-2832	Georgia	41.82	44.82	0.10	13	TauL3	-	-	-	KYOTO/NBRP

Table S2 Germinability and seedling growth ability in the three lineages of *Ae. tauschii* under different NaCl-induced-stress conditions

Trait	Treatment (% NaCl)	Lineage	No. of observed accessions	No. of unobserved accessions	Mean germinated seed number /first leaf lengths (mm)	Standard deviation	*P*-value for the TauL1 vs. TauL2 comparison
Germinability	0.0	TauL1	133	0	8.4	1.0	0.00
		TauL2	68	0	8.0	1.2	
		TauL3	5	0	8.4	0.5	
	0.5	TauL1	133	0	7.9	1.4	0.00
		TauL2	68	0	6.5	1.9	
		TauL3	5	0	7.6	0.5	
	1.0	TauL1	133	0	6.5	1.8	< 0.00
		TauL2	68	0	2.9	2.1	
		TauL3	5	0	4.4	0.9	
	1.5	TauL1	133	0	3.1	2.1	< 0.00
		TauL2	68	0	0.4	0.8	
		TauL3	5	0	0.2	0.4	
Seedling growth ability	0.0	TauL1	133	0	83.3	17.7	0.59
		TauL2	68	0	85.0	22.6	
		TauL3	5	0	65.8	10.9	

0.5	TauL1	132	1	70.1	12.4	0.20
	TauL2	68	0	67.4	15.4	
	TauL3	5	0	56.9	6.5	
1.0	TauL1	131	2	47.5	10.2	<0.00
	TauL2	60	8	37.7	14.1	
	TauL3	5	0	32.8	3.7	
1.5	TauL1	119	14	28.0	8.0	0.03
	TauL2	14	54	21.5	9.5	
	TauL3	1	4	20.0	N/A	

N/A denotes that the value is not available.

Table S3 Comparison of TauL1-germinability PC and TauL1-seedling PC values

Trait	Sublineage/group	No. of accessions	Mean	Standard deviation
TauL1-germinability PC*	TauL1a	50	0.19[a]	0.35
	TauL1b	74	-0.15[a]	0.29
	TauL1x	9	0.19[a]	0.82
TauL1-seedling PC**	TauL1a	50	-0.35	0.20
	TauL1b	72	0.33	0.16
	TauL1x	9	-0.73	0.46
TauL1-seedling PC (within TauL1b)***	HighQ2	26	1.23[a]	1.11
	LowQ2	41	-0.25[b]	1.74
	Q3	5	0.38[ab]	1.25

*Means with a common superscript are not significantly different (Tukey-Kramer test, $P > 0.05$)

**Significantly different mean values were only found between TauL1a and TauL1b (Dunnett T3 multiple comparisons, $P = 0.013$).

***Means with a common superscript are not significantly different (Tukey-Kramer test, $P > 0.05$)

Table S4 Comparison of germinability and seedling growth ability between the TauL1 sublineage groups

Trait	Sublineage groups	No. of accessions	Mean	Standard deviation
Germinability*	TauL1a	50	8.6[a]	0.8
	TauL1b	74	8.3[a]	1.1
	TauL1x	9	8.3[a]	0.7
Seedling growth ability**	TauL1a	50	78.2	13.7
	TauL1b	74	87.4	19.7
	TauL1x	9	77.8	10.5

* Means with a common superscript are not significantly different (Steel-Dwass test, $P > 0.05$)

** Significantly different mean values were only found between TauL1a and TauL1b (Dunnett T3 multiple comparisons, $P = 0.004$).

Table S5 Pairwise F_{ST} values between the HighQ2, LowQ2, and Q3 groups of TauL1b based on the polymorphisms at 118 molecular marker loci

Group	HighQ2	LowQ2	Q3
HighQ2		0.001	0.001
LowQ2	0.151		0.001
Q3	0.452	0.333	

The molecular marker dataset was taken from Matsuoka et al. (2015) (BMC Evol. Biol. 15: 213). F_{ST} values are below the diagonal. Permutation *p*-values based on 999 permutations are shown above the diagonal.

Table S6 Number of long first-leaf and short first-leaf accessions sampled in northern and southern habitats

Habitat	TauL1b group	Long first-leaf accession	Short first-leaf accession
North (latitude > 37.5˚)	HighQ2	0	2
	LowQ2	4	11
	Q3	0	0
South (latitude ≤ 37.5˚)	HighQ2	23	1
	LowQ2	11	15
	Q3	3	2

Table S7 Number of long first-leaf and short first-leaf accessions sampled in three different habitats defined by their characteristic edaphic conditions

Habitat edaphic condition	TauL1b group	Long first-leaf accession	Short first-leaf accession
Increased salinity and reduced available water capacity (IR)	HighQ2	12	0
	LowQ2	1	2
	Q3	0	0
Reduced salinity and reduced available water capacity (RR)	HighQ2	9	1
	LowQ2	6	10
	Q3	0	0
Reduced salinity and increased available water capacity (RI)	HighQ2	0	2
	LowQ2	4	12
	Q3	3	2

Six long first-leaf and two short first-leaf accessions were excluded due to missing soil salinity values.

Table S8 Linear regressions of *Ae. tauschii*'s reproductive traits on TauL1 seedling PC values or mean first leaf lengths under control (distilled water) conditions

Trait	Source	Phenotype group	*N*	R^2	Parameter estimate	Standard error	*t* statistic	*P*
Flowering time	TauL1 seedling PC value	Long first leaf	41	0.00	0.03	0.73	0.04	0.97
		Short first leaf	31	0.18	-4.42	1.75	-2.52	0.02
Flowering time	Mean first leaf (distilled water)	Long first leaf	33	0.05	0.08	0.07	1.29	0.21
		Short first leaf	39	0.02	-0.18	0.18	-0.96	0.34
Number of spikelets per spike	TauL1 seedling PC value	Long first leaf	39	0.02	0.16	0.22	0.75	0.46
		Short first leaf	30	0.00	-0.01	0.37	-0.04	0.97
Number of spikelets per spike	Mean first leaf (distilled water)	Long first leaf	32	0.01	0.01	0.02	0.64	0.53
		Short first leaf	37	0.01	-0.02	0.04	-0.57	0.57

See Fig. 6 for regression graph.
